# Association of Oxidative Stress on Pregnancy

**DOI:** 10.1155/2020/6398520

**Published:** 2020-09-15

**Authors:** Kinga Toboła-Wróbel, Marek Pietryga, Piotr Dydowicz, Marta Napierała, Jacek Brązert, Ewa Florek

**Affiliations:** ^1^Department of Obstetrics and Female Health, Chair of Gynaecology, Obstetrics and Gynaecological Oncology, Poznan University of Medical Sciences, 60-535 Poznan, Poland; ^2^Ultrasound and Prenatal Diagnostic Laboratory, Gynaecology and Obstetrics Hospital, Poznan University of Medical Sciences, 60-535 Poznan, Poland; ^3^Laboratory of Environmental Research, Department of Toxicology, Poznan University of Medical Sciences, 60-631 Poznan, Poland

## Abstract

The pathophysiological mechanism underlying pregnancy complications such as congenital malformations, miscarriage, preeclampsia, or fetal growth restriction is not entirely known. However, the negative impact of the mother's body oxidative imbalance on the fetus and the course of gestation is increasingly discussed. This article is an integrative review of some original studies and review papers on the effects of oxidative stress on the adverse pregnancy outcomes mainly birth defects in fetuses. A systematic search for English language articles published from 2010 until 2020 was made, using MEDLINE data. Additionally, we analyzed the Cochrane and Scopus databases, discussions with experts, and a review of bibliography of articles from scientifically relevant and valuable sources. The main purposes are to assess the contribution of the existing literature of associations of oxidative stress on the etiology of the abovementioned conditions and to identify relevant information and outline existing knowledge. Furthermore, the authors aim to find any gaps in the research, thereby providing grounds for our own research. The key search terms were “oxidative stress in pregnancy,” “oxidative stress and congenital malformations,” and “oxidative stress and adverse pregnancy outcomes.” Studies have confirmed that oxidative stress has a significant impact on pregnancy and is involved in the pathomechanism of adverse pregnancy outcomes.

## 1. Introduction

Over the past few years, more and more attention has been devoted to issues of environmental impact, lifestyle, and comorbidities on the body's oxidative balance and its possible impact on abnormalities related to fetal development and pregnancy outcome. The term oxidative stress refers to the imbalance between the production of reactive oxygen species (ROS) and the ability of antioxidant mechanisms to neutralize them. It may be the result of an increase in ROS generation and/or a weakening of antioxidant defense [1, 2]. Analyzing the available literature of the last decade, it has been found that the topic of oxidative stress in pregnancy is becoming more common and knowledge on this subject is expanding.

The causative factor of the analysis is the previously unexplained etiology of most frequent pregnancy complications, such as miscarriages, FGR, or preeclampsia, in particular congenital defects. The main purposes are to assess the contribution of existing literature of the association of oxidative stress on the etiology of the abovementioned conditions and to identify relevant information and outline existing knowledge. Furthermore, the authors aim to find any gaps in the research, thereby providing grounds for our own research.

The article is an integrative overview of the original studies and review papers concerning the role of oxidative stress in the pathogenesis of pregnancy complications with particular emphasis on congenital defects and chromosomal aberrations. A review of articles published from 2010 to 2020 in English has been carried out by searching the MEDLINE database using the terms “oxidative stress in pregnancy,” “oxidative stress and congenital malformations,” and “oxidative stress and adverse pregnancy outcomes.” Additional search has been carried out using the analyses of the Cochrane and Scopus databases, discussions with experts, and a review of bibliography of articles from scientifically relevant and valuable sources.

The research strategy is attached as Table 1.

To sum up, the main question to which the authors seek the answers to in this article is: “Does oxidative stress have a significant impact on the course of pregnancy and the occurrence of birth defects?”

## 2. The Body's Prooxidative and Antioxidant System

Reactive oxygen species (ROS) are products of the body's incomplete reduction of oxygen molecules. They oxidize fats, proteins, and DNA and thus can contribute to tissue damage. Toxic oxidation reaction products exert a cytostatic effect on the cell, damage cell membranes, and activate mechanisms of apoptosis. The ROS includes superoxide anion radical (O2∙), hydroxyl radical (OH∙), hydroperoxide radical (HO2∙), peroxide radical (ROO∙), and alkoxy radical (RO∙) and others: hydrogen peroxide (H2O2), hypochlorous acid (HOCl), and subbromic acid (HOBr) [3–5].

The body has also developed an antioxidant system consisting of preventing, combating, eliminating, and repairing the effects of ROS reactions with biological molecules to defend against them. Due to the mechanism of action, antioxidants can be divided into enzymatic: superoxide dismutase (SOD), catalase (CAT), glutathione peroxidase (GSH-Px), ceruloplasmin, heme proteins, thioredoxin (TRX), and paraoxonase (PON1) and nonenzymatic: glutathione (GSH), vitamin E, vitamin C, albumin, bilirubin, uric acid, creatinine, cysteine, carotenoids, flavonoids, coenzyme Q (reduced), metal ion binding proteins (ferritin, metallothioneins), and blood plasma proteins (transferrin, ceruloplasmin, albumin) [6–10].

The list of disease entities in which the causative and negative effects of oxidative stress has been proven to increase as research into the mechanisms of its action develops [11–16].

The studies carried out so far concern the occurrence of oxidative stress in the body by increased concentrations of lipid peroxidation products and reduced activity of antioxidant enzymes or other antioxidants. [17–20] These reduced values of oxidative enzymes can disturb the body's prooxidative-antioxidative homeostasis by participating in the pathogenesis of diseases, and increased values of oxidative stress can be a symptom of an adaptive reaction and an attempt to alleviate the effects of these pathological changes [21, 22].

**Figure 1:**
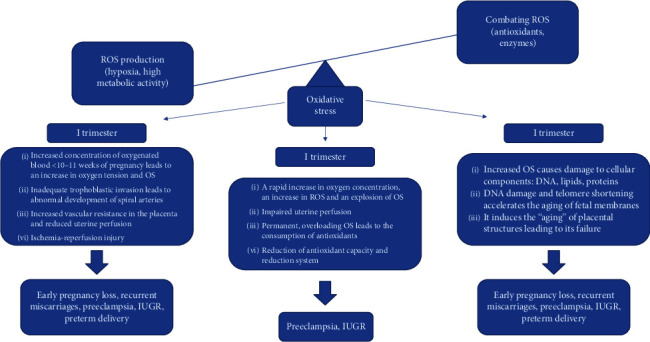
Impact of oxidative stress on function and abnormal pregnancy development [23].

Pregnancy is a time when the body's oxidative imbalance negatively affects its development and causes various types of complications depending on the stage of its development. This mechanism is described in Figure 1.

The relationship between abnormal metabolism, disturbances in ROS production, the body's oxidative balance, and diseases can be the cause, stage, or effect of a disease process. Currently, it is not possible to clearly define the direction of changes, but it is increasingly emphasized as the role of causative factor [23].

## 3. Oxidative Stress in Pregnancy with Normal Pregnancy Outcome

During physiological pregnancy, the development of fetal tissues and organs requires the supply of an adequate amount of nutrients and oxygen, and its reactive forms produced in the body of the mother and the fetus affect the replication, differentiation, and maturation of the developing cells. Their balanced activity and maintaining the balance of oxidative processes are necessary factors for the proper development and functioning of the body [24].

During pregnancy, numerous anatomical, physiological, and metabolic changes occur in the mother's body. According to the researchers, it is assumed that they support the production of ROS, especially in the second half of pregnancy. This is mainly due to an increasing basic metabolism and “consumption” of oxygen and the use of fatty acids as the primary source of energy for most maternal retroplacental tissues. The last trimester of pregnancy is a special period of increasing insulin resistance, fat catabolism, and the release of free fatty acids. These processes lead to increased production of hydrogen peroxide [25].

The placenta, filled with mitochondria, is the main source of prooxygenates, the so-called ROS “factory.” The superoxide anion radical (O- ∙ 2) produced in large quantity is a source of the formation of further active forms of oxygen, i.e., hydrogen peroxide and hydroxyl radical. Their production increases with the development of pregnancy, which is mainly associated with an increase in placental mass. Nitric oxide is also synthesized by macrophages mainly in the placenta.

In a correctly developing pregnancy, the phenomenon of the mother's immune tolerance to the fetus' antigens, which allows the fetus to develop in the uterus despite the pregnant woman's ability to reject the foreign antigen, is an extremely important aspect. The main assumptions of this phenomenon are partial inhibition of the mother's immune system during pregnancy, insufficiently strong presentation of fetal antigens, the placenta as an important element separating the woman from the fetus, and changing the direction of the organism's specific response to the Th2 response instead of Th1 (cytokines produced mainly by nonspecific system cells—NK type (natural killer)). Immunological tolerance is therefore formed by the cells of the implanted trophoblast, the mother's immune system, and the microenvironment of the implanted embryo—a decidual cell. It enables the correct implantation and development of the embryo and functioning of the placenta. Due to the reduction of the immune system works in a properly functioning pregnant organism, the production of ROS is lowered.

Erythropoiesis increases, erythrocyte life expectancy decreases, and increased iron delivery to the fetus increases its availability by catalyzing the increase in the generation of large amounts of reactive hydroxyl (∙OH) radicals in the Fenton reaction [26].

Numerous studies prove that oxidative stress, i.e., excessive and unbalanced ROS production, has adverse effects on pregnancy, pregnant health, and fetal development. It is the cause of incorrect implantation of embryos, miscarriages, premature births, low birth weight, and malformations. It also weakens pregnant immunity and respiratory adaptation of newborns immediately after birth. The main reason for these disorders is the insufficient supply of nutrients and oxygen to the fetus resulting mainly from hypoplasia and abnormal placental function [23–25].

Comparative studies of pregnant and nonpregnant patients [27] showed that total plasma antioxidant status (TAS) in the first trimester of pregnancy is significantly lower. In the second and third trimesters of pregnancy, total plasma antioxidant capacity (TAC) increases, and in the last week of pregnancy reaching values similar to those observed in nonpregnant women. After delivery, this rate increases to the eighth week after delivery, and these changes are proportional to changes in plasma uric acid.

Studies by other scientists indicate that the reason for lower TAS values in pregnancy is the reduction in serum albumin, bilirubin, and vitamin E levels [28]. It was also found that during a properly developing pregnancy, plasma superoxide dismutase activity decreases [29].

Physiologically, during pregnancy, it increases the concentration of triglycerides, total cholesterol, and low-density lipoprotein (LDL) cholesterol levels in plasma as well as markers of oxidative stress, which is associated with an increase in lipid peroxides after 25 weeks of pregnancy. Therefore, the natural indicator of oxidative stress and the degree of lipid peroxidation is increasing concentration of malondialdehyde in the plasma of pregnant women.

There have also been reports of the effect of a diet with vitamins, antioxidants, and minerals on the value of total antioxidant status in pregnant patients [28–30].

The potential influence of antioxidant and vitamin supplementation on the course of pregnancy is shown in Table 2.

## 4. Oxidative Stress in Pregnancy Completed with Birth Defects (Table 3)

The problem of birth defects in the fetus concerns about 4-6% of live-born newborns and the majority of miscarriages in the first trimester of pregnancy, the exact number of which cannot be determined. They are also the most common cause of infant mortality and disability in the 21st century. The most commonly known causes of congenital defects are genetic factors. However, approximately 50% of congenital anomalies cannot be linked to a specific cause.

Oxidative stress, which is primarily the result of excessive production of oxygen peroxidation products, mainly in mitochondria, attacks newly growing cells. It damages their structures probably already at an early stage of embryogenesis. Determinants of the production of an increased number of ROS and disorders caused by them can be environmental pollution, chronic stress, low levels of physical activity, teratogenic effect of drugs and chemicals, or improper nutrition. It causes abnormalities in the structure of DNA that can lead to early miscarriages, preeclampsia, fetal growth restriction, fetal abnormalities, and birth defects [31–33].

Researchers found elevated levels of oxidative stress markers in the blood serum of pregnant mothers during the first prenatal examination at 11-14 gestational week, who had a high (<1: 300 according to Fetal Medicine Foundation) risk of fetal malformations. Then, after further research and detailed analysis, they confirmed the significant difference between the levels of oxidative stress in patients with healthy and diseased fetuses—complicated by chromosomal aberrations and other malformations [34].

Due to the damage of the DNA structure by an unbalanced antioxidative and oxidative level in the body, more attention is paid to the role of ROS in the etiopathogenesis of genetic defects. Oxidative stress damaging the structure of a deoxyribonucleic acid molecule leads to chromosomal aberrations. Pagano and Castello [35] showed characteristic changes in vivo of mitochondrial function, leading to increased ROS concentration in the cell as changes characteristic of trisomy 21. Analyses of several markers of oxidative stress, in pregnant blood, amniotic fluid, and fetal tissues clearly showed its association with Down syndrome. Observed changes had a significant share in the damage of various tissue enzymes. It can be presumed that they played a role in the pathogenesis of this abnormality [36].

The metabolomic analysis of blood serum from patients with Down syndrome in the fetus, in the first trimester of pregnancy, showed differences in the levels of 2-hydroxybutyrate in comparison with patients with healthy fetuses. The level of substance, which is physiologically involved in the defense against oxidative stress, was lower in the study group, proving the oxidative imbalance in the pregnant body with fetal trisomy 21 [37].

There was also a conducted research about the rate of fibroblast proliferation and its major regulators, such as Rcan1 or telomere length, for assessing the oxidative balance of these cells in fetuses with Down syndrome. RNA expression and activity of the main antioxidant enzymes in the study group were analyzed. The thesis regarding the effect of oxidant-antioxidant imbalance on the generation of genetic disorders such as trisomy 21 was confirmed. An increased GSSG/GSH ratio and high concentrations of protein peroxidation products in fibroblasts have been demonstrated. The obtained values correlated with the reduced antioxidant capacity of cells. The results obtained showed reduced levels of antioxidants that cooccurred with increased Rcan1 levels and telomere shortening, responsible for increased oxidative stress and cell cycle disorders of fibroblasts of fetal's with Down syndrome [38].

It has also been proven that the amniotic fluid, in which fetal cells with Down's syndrome are present, differs in oxidative status depending on the severity of the lesions and abnormalities that occur—especially nerve cell damage. Increased levels of oxidative stress, as indexed by increased protein oxidation, lipid peroxidation, reduction of glutathione (GSH) and thioredoxin levels, and induction of the heat-shock protein (HSP) response were associated with worse prognosis as to survival and normal development [39].

A relationship between oxidative stress and the occurrence of such abnormalities in the genome structure as Ataxia-telangiectasia (A-T), Bloom syndrome (BS), or Nijmegen syndrome (NBS) has also been demonstrated. High levels of reactive oxygen species (ROS) may be a major phenotypic hallmark in these diseases. The observed damages and other abnormalities, such as changes in the ultrastructure and function of cells, prove that we can consider analyzed diseases as mitochondrial. However, more research is needed to confirm whether antioxidants and free radical scavengers can improve the condition or extend the survival of patients [40].

Scientists conducting research on the pathogenesis of Treacher Collins syndrome have demonstrated the negative effects of ROS on developing progenitor neural crest cells. Depending on the degree of DNA damage, fetal death or abnormal development of the facial cranium occurred. The confirmation of the theory was the absence of a defect in the progeny of mammals undergoing antioxidant supplementation. High levels of ROS were the causative agent of facial cranial malformations [41].

The relationship of oxidative stress with the occurrence of cardiac malformations was also analyzed. After investigating the levels of oxidative stress markers in newborns with cyanotic and noncyanotic congenital heart defects, elevated levels of oxidative stress and reduced levels of antioxidants were found in sick patients. In addition, studies with increased levels of homocysteine and reduced levels of vitamin B12, glutathione as an antioxidant, and folate in pregnant patients with fetal heart disease have been published. Increased markers of oxidative stress in the study group suggests its participation in the etiopathogenesis of cardiac developmental abnormalities [42].

Analysis of the oxidative balance in fetuses with trisomy 21 and coexisting congenital heart defects showed an impairment of the mitochondrial respiratory chain, inhibition of Complex I, and consequently increased production of ROS. The analysis of the heart cell transcriptome with abnormalities showed that the function of genes responsible for the regular work of mitochondria is significantly reduced compared with the control group, suggesting their participation through oxidative stress in the manifestation of the defect [43].

Researchers also investigated the oxidative balance of the fetuses of mothers with preexisting diabetes who had heart defects (CHD), such as a defect in the ventricular or atrial septum, valve defects, or abnormal ventricular outflow tracts. Mouse models have shown that oxidative imbalance is a major determinant of CHD. Concentrations of nitric oxide (NO) and reactive oxygen species dependent on endothelial nitric oxide synthase (eNOS) are crucial for creating the right structures of the heart muscle, regulating various cellular and molecular processes. As a result, eNOS deficiency causes oxidative stress, CHD, and coronary artery malformations [44].

Fetal exposure of a pregnant woman to heavy metals, such as lead and aluminum, has also been shown to be negative by disturbing the prooxidative-antioxidant balance. It is associated with an increase in MDA concentration and a decrease in the level of antioxidants such as superoxide dismutase (SOD) and glutathione peroxidase (GPx) in fetal cord blood serum with congenital heart disease [45].

The association of oxidative stress on the development of congenital malformations of the central nervous system is also increasingly well known. They belong to the common and one of the most serious birth defects in fetuses. Numerous studies have been carried out confirming the relationship between oxidant-antioxidant imbalance and abnormal development of the nervous system. A negative effect of oxidative stress on cell differentiation and CNS development has been demonstrated [46, 47]. Increased ROS concentrations have been documented in such anomalies as holoprozencephalia or myelomeningocele. Reduced levels of antioxidants such as glutathione and catalase as well as increased levels of malondialdehyde in fetal amniotic fluid with central nervous system development disorders have also been shown [48].

Publications on the pathophysiology of oxidative stress in congenital malformations are shown in chronological order in Table 3.

## 5. Oxidative Stress in Pregnancy Complicated by Other Abnormalities

Researches to date confirm that the balance of oxidative-antioxidative processes is important for proper implantation and embryo development, while uncontrolled production of oxygen peroxidation products can lead to embryo resorption, embryopathy, development of preeclampsia, or degeneration of the placenta resulting in inhibition of fetal growth, low birth weight, or even premature delivery. There are also reports confirming that the occurrence of some pregnancy complications, such as fetal growth restriction (FGR) or preeclampsia, may be associated with antioxidant deficiency [49, 50].

The proper development of the placenta, from conception, requires balanced oxygen metabolism. The relatively low oxygen pressure in the initially developing chorion is intended to prevent excessive ROS production, protecting the embryo and fetus against the harmful and teratogenic effects of free radicals [51].

The researchers showed differences in levels of oxidative stress markers in the placenta, as well as increased apoptosis and reduced trophoblast cell proliferation in patients who had a miscarriage in the first trimester of pregnancy compared to normal developing pregnancies. In these patients, transcriptomic studies showed reduced expression of the genes responsible for mitochondrial function in placental villi. The exact associations of oxidative stress on the placenta in early pregnancy loss remain unclear. However, it is known that in placental cell cultures from patients with early miscarriages, the level of stress in the structure of the endoplasmic reticulum is higher, and the response of endoplasmic reticulum (UPR) chaperone proteins, which are a repair mechanism, is less effective [52].

Numerous studies for gestational hypertension and preeclampsia have confirmed that the reactive oxygen species are working efficiently. The authors point to the significant importance of reducing the levels of such oxidants, e.g., vitamin E or C in this type of pregnancy pathologies. A significantly lower antioxidant effect of trolox—a substance derived from vitamin E on lipid peroxidation processes in the placenta of women suffering from preeclampsia—has been proven. And by analyzing endothelin-1 and lipid peroxides in the serum of pregnant women with hypertension, a decrease in the level of abnormal oxidation reaction substances in pregnant women with hypertension supplementing vitamin E was shown [53].

Fetal growth restriction has also been analyzed for the relationship between its occurrence and oxidative stress. It has been shown that damage resulting from ROS activity occurs mainly in membrane lipids, proteins, and nuclear and mitochondrial DNA. Plasma and tissue malondialdehyde (MDA) levels were used for this analysis, which is an indicator of lipid peroxidation and oxidative stress. MDA and xanthine oxidase (XO, ROS-producing enzyme) levels were higher in maternal plasma, umbilical cord plasma, and placental tissues of patients with IUGR-complicated pregnancy. 8-Hydroxy-2′-deoxyguanosine (8-OHdG) is one of the more commonly used oxidation markers as the effect of ROS on tissues. Its levels and redox-1 factor (redox regulator, responsible for repairing damaged DNA) are much higher in the placenta of patients in pregnancy complicated by FGR compared to a properly developing pregnancy. These patients also demonstrated different levels of antioxidant enzymes, resulting in an increase in SOD and GSH-Px activity and a decrease in CAT activity. Analysis of placental structure in patients with intrauterine growth retardation confirmed the theory of aging associated with prooxidative-antioxidant imbalance and the effect of ROS on trophoblast cells [23].

After analyzing several available publications on preterm labor, a relationship between its occurrence and the oxidative balance of the pregnant body was also demonstrated. Patients who gave birth prematurely (before 37 weeks of pregnancy) had increased values of the total oxidative capacity of the body and lipid peroxidation products, which were the most frequently analyzed markers. It was also associated with reduced levels of the mother's total antioxidant capacity [54].

Publications on the pathophysiology of oxidative stress in pregnancy abnormalities are summarized in chronological order in Table 4.

## 6. Summary

The integrative review has shown that reactive oxygen species (ROS) play an important role in the regulation of cellular signaling and genetic expression. Based on the results of previous studies between 2010 and 2020 and widely documented muta- and carcinogenic properties of oxidative DNA damage, we analyzed the relationship between oxidative stress and the occurrence of congenital defects, which in our opinion seems to be a potential causative factor. We also analyzed the contribution effects of oxidative stress on other complications of pregnancy, such as FGR, diabetes, or preeclampsia.

In conclusion oxidative stress is unquestionably linked to abnormalities in both the pregnant mother and fetal development. However, it is not yet possible to clearly establish and confirm that it is the causative factor, due to numerous doubts and the lack of a specific mechanism of action. The final statement requires further detailed research, especially of clinical significance.

## Figures and Tables

**Table 1:** Search strategy.

Search strategy	
Purposes	(i) Assess the contribution of existing literature of association of oxidative stress on the etiology of pregnancy complications (miscarriages, congenital malformations/chromosomal abnormalities, FGR, preeclampsia) (ii) Identify relevant information and outline existing knowledge (iii) Identify any gap in the research thereby providing a rationale for our own studies

Research question	(i) Does oxidative stress have a significant impact on the occurrence of disorders during pregnancy, and in particular birth defects in the fetus?

Keywords	(i) “Oxidative stress in pregnancy” (ii) “Oxidative stress and congenital malformations” (iii) “Oxidative stress and adverse pregnancy outcomes”

**Table 2:** Effect of vitamins, micronutrients, and antioxidants on pregnancy outcome.

References	Markers analyzed	Disease units analysed	Commentary
Dennery [28] (USA)	Lazaroid—new class of lipophilic steroids Polyunsaturated fatty acids (PUFAs) Vitamin C and E—antioxidant	Vascular dysfunction of the mother Hypertension IUGR	Administration of lazaroid = ↓ risk in offspring. Melatonin solution administration = ↓ risk of oxidative DNA damage (↓ IUGR cases) PUFAs administration = ↑ ROS in oocytes = impaired embryonic development Combined vitamin C and E supplementation = not useful in preventing preeclampsia

Mistry et al. [30] (UK)	Selenium—component of glutathione peroxidase antioxidant enzymes, thioredoxin reductases, and selenoprotein-P Copper and zinc—essential elements of Cu/Zn superoxide dismutase (SOD) Manganese—cofactor for the antioxidant manganese superoxide dismutase (Mn-SOD) Vitamin C—antioxidant Vitamin E—antioxidant	Reccurent pregnancy loss (RPL) Postpartum thyroid dysfunction (PPTD) Preeclampsia (PE) pregnancy-induced hypertension (PIH) Prolonged labour Fetal growth restriction (FGR) Embryonic or fetal death Perinatal death Preterm prelabour rupture of membranes (PPROM)	In mother's serum: ↓ selenium concentrations = ↑ risk of RPL Selenium supplementation (women positive for thyroid peroxidase antibodies) = ↓risk for PPTD, PE, and PIH Copper itself = acting as a prooxidant, associated in Cu/Zn SOD = acting as an antioxidant Zinc supplementation = ↓ risk of PIH and low birth weights ↓ manganese concentrations = ↑ risk of FGR Vitamin C and E supplementations = no impact on PE and PIH, ↑ risk of fetal loss, perinatal death and PPROM

Pasiński et al. [53] (Poland)	Vitamin C—antioxidant Vitamin E—antioxidant	Preeclampsia (PE) pregnancy-induced hypertension (PIH)	Vitamin C and E supplementations = ↓ lipid peroxides and ROS

**Table 3:** Current studies concerning the influence of oxidative stress on congenital malformations.

References	Markers analyzed	Abnormalities investigated	Commentary
Hernández-García et al. [29] (Mexico)	Dichlorofluorescin (DCFH) 3′-(p-Aminophenyl) fluorescein (APF) 3′-(p-Hydroxyphenyl) Fluorescein (HPF) Hydroethidine (HE) Dihydrorhodamine (DHR) Mono- and diboronated sensors Boron dipyrromethene difluoride (BODIPY-) based dyes Hydrocyanines	Cell development—early embryogenesis	Review: uncertain and unspecific evidence on the role of ROS in development. Assessment of recent methods to detect ROS in vivo—markers of cellular ROS production in embryos

Dennery [28] (USA)	Reactive oxygen species (ROS)	Human development	Review: impact of redox state on fetal development and placenta High ROS levels cause impairment fetal development and placental function Possible therapeutic interventions with antioxidant: Vit C and E—uncertain

Bahado-Singh et al. [37] (USA)	Male gender Oxidative stress 8-Hyroxy-2-deoxyguauosine, glutathione, vitamin A	Cyanotic congenital heart disease (CCHD), anencephaly, spina bifida, congenital diaphragmatic hernia (CDH), omphalocele, gastroschisis, limb defects, cleft lip with or without cleft palate (CL/P), and isolated cleft palate	Increased OS in males Development of CCHD, omphalocele, neural tube, and facial cleft is linked to increased OS (↑8-hyroxy-2-deoxyguauosine, glutathione) (↓vitamin A)

Perluigi et al. [39] (Italy)	Protein carbonylation Protein-bound HNE Reduced glutathione (GSH) Heat shock proteins (HSPs) Thioredoxin (Trx)	Down syndrome	Oxidative damage—early event in DS pathogenesis ≥ deleterious DS phenotypes (abnormal development, neuropathology)

Piccoli et al. [44] (Italy)	Mitochondrial respiratory activity Reactive oxygen species (ROS)	Down syndrome Congenital heart defects	Mitochondrial disfunction = ↑ OS ≥fibroblast ≥congenital heart defects in DS

Bahado-Singh et al. [37] (USA)	3-Hydroxybutyretate (oxidative stress marker) 3-Hydroxyisovalerate	Down syndrome	Oxidative stress is thought to be one of the most likely causes of neurotoxicity in DS

Zong et al. [33] (China)	Polymorphisms in glutathione S-transferases (GSTs) GSTA1-69C/T	Recurrent spontaneous abortion (RSA)	No significant association between RSA and GSTs polymorphisms

Gimeno et al., [38] (Spain)	Telomere length (TL) Peroxide levels GSSH/(glutathione) GSH ratio Cu/ZnSOD, MnSOD activity Catalase activity Glutathione peroxidase activity	Down syndrome	Alteration of SOD1gene expression, Cu/Zn SOD protein levels and other antioxidant enzymes (thioredoxin 1) ≥poor proliferative capability of tissues in DS (telomeric attrition, increased expression of Rcan1) - > ↑OS - > pathophysiology of DS

Mukhopadhyay et al. [42] (India)	Malondialdehyde (MDA) Protein carbonyl (PC) Vitamin C Reduced glutathione (GSH)	Tracheesophageal fistula (TEF) Anorectal malformation (ARM) Intestinal atresia (IA)	↑ MDA, PC (products of lipid and protein oxidation) = pathophysiology involves OS Treatment with antioxidants = useful as a preventive therapy

Sakai et al. [41] (Japan)	Reactive oxygen species (ROS) *N*-Acetyl-cysteine (NAC)	Treacher Collins syndrome	Tcof1 haploinsufficiency results in OS-induced DNA damage and neuroepithelial cell death Maternal treatment with antioxidants minimizes cell death in the neuroepithelium and substantially ameliorates/prevents the pathogenesis of craniofacial anomalies in *Tcof1*^+/−^ mice

Yuan et al. [48] (China)	8-Hydroxy-2′-deoxyguanosine (8-OHdG), protein carbonyl (PC), and 8-iso-prostaglandin F2*α* (8-iso-PGF2*α*)	Neural tube defects (NTDs)	↑ 8-OhdG—without folic acid supplements during the periconceptional period ↑ 8-OhdG—pregnancies affected by NTDs

Moore et al. [55] (Turkey)	Reactive oxygen species (ROS)	Congenital malformations	OS = harmful radicals attacking biological molecules: DNA, lipids, proteins

Ozsurekci et al. [56] (Japan)	Reactive oxygen species (ROS) TCOF1 gene	Treacher Collins syndrome (TCS)	Review: role of Tcof1 mutation in embryonic craniofacial development Genetic and environmental factors ≥severity of craniofacial abnormalities, prospect for prenatal prevention of craniofacial anomalies

Maciejczyk et al. [40] (Poland)	Total antioxidant capacity (TAC) Malondialdehyde (MDA)	Ataxia-telangiectasia (A-T), Bloom syndrome (BS) Nijmegen breakage syndrome (NBS)	A-T, BS, and NBS may be considered mitochondrial diseases. Excess activity of antioxidant enzymes and an insufficient amount of low molecular weight antioxidants indicate new pharmacological strategies for treatment.

Pietryga et al. [34] (Poland)	Glutathione (GSH) Glutathione S-transpherase (GST) S-Nitrosothiols (RSNO) Trolox equivalent antioxidant capacity (TEAC) Total protein (TP) Nitrites	Chromosomal aberrations Congenital malformations	↑ TP, GST, TEAC, and ↓ GSH correlated with the risk of chromosomal aberrations and congenital malformations

Liu et al. [43] (China)	Lead (Pb) Aluminum (Al) Malondialdehyde (MDA) Supeoxide dismutase (SOD) Glutathione peroxidase (GSH-Px)	Congenital heart disease	Heavy metals ≥↑ oxidative stress ≥congenital heart disease

Cim et al. [46] (Turkey)	Glutathione (GSH) Catalase (CAT) Malondialdehyde (MDA)	Congenital malformations of the central nervous system	↑ MDA ↓ GSH and CAT = ↑ OS in amniotic fluid—associated with neural tube defects

Laforgia et al. [36] (Italy)	Oxidative stress Reactive oxygen species (ROS) Antioxidants	Down syndrome Heart malformation Neural tube effect	Review: fetal tissue—sensitive to oxidative damage OS + impaired antioxidant activity = congenital malformations Antioxidants therapeutic approaches

Lin et al. [47] (China)	Benzo[*α*] pyrene (BaP) Reactive oxygen species (ROS) Superoxide dismutase (SOD) Glutathione peroxidase (GPx) Catalase (CAT) 8-hydroxy2′deoxyguanosine Total antioxidant capacity (TAC) Malondialdehyde (MDA)	Neural tube defects	BaP exposure ≥↑ OS, apoptosis ≥NTDs Protective effect of vitamin E

Engineer et al. [45] (Canada)	Review Nitric oxide (NO) Nitric oxide synthase (eNOS) ROS 8-hydroxyguanosine (8-OHG)	Congenital heart disease	eNOS and NO—critical for property morphogenesis of all major components of the developing heart ↑ ROS—nonspecific damage and permanent functional changes 8-OHG—RNA damage causative factor Hyperglycemia = ↑ROS Treatment with Vit E and C = ↓ rate and severity of CHD

**Table 4:** Current studies concerning the influence of oxidative stress on adverse pregnancy outcomes.

References	Markers analyzed	Abnormalities investigated	Commentary
Bogavac et al. [49] (Serbia)	Reduced glutathione (GSH) Supeoxide dismutase (SOD) Glutathione peroxidase (GSH-Px) Glutathione reductase (GSHR) Glutathione S-transferase (GST) Xanthine oxidase (XOD) Lipid peroxidation (LP)	Pregnancy-induced hypertension (PIH) Gestational diabetes mellitus (GDM) Bacterial vaginosis	Different concentrations in a variety of conditions Parameters of oxidative stress in the amniotic fluid could be altered in certain pathological conditions
Clerici et al. [31] (Italy)	Total antioxidant capacity (TAC) Thiolyte capacity Prooxidant capacity	Preeclampsia (PE) Pregestational diabetes (DM) Preterm birth (PTB)	↑ OS = pathological pregnancies ↑ prooxidant capacity ↓ TAC and thiolyte capacity in pathological pregnancies
Yiyenoglu et al. [32] (Turkey)	Total antioxidant capacity (TAC) Total oxidant level (TOL) Oxidative stress index (OSI)	Recurrent pregnancy loss (RPL)	↑ TOL and OSI ↓ TAC in RPL group OS plays a central role in the etiopathogenesis of RPL
Marseglia et al. [50] Italy	Oxidative stress (OS) Reactive oxygen species (ROS)	Recurrent pregnancy loss Preeclampsia preterm premature rupture of membranes	Review: free radical theory of aging, abnormal placentation ≥↑OS ≥recurrent abortions (RPL), preeclampsia, FGR, PTB, pPROM
Ramkumar [57] (USA)	Reactive oxidative stress	Preterm birth (PTB) Premature rupture of membranes (pPROM) Inflammation	OS affects cell aging, inflammation, pPROM, and consequently PTB by p38 mitogen-activated kinase (p38MAPK) pathways
Jauniaux et al. [51] (United Kingdom)	Pathophysiology review	Miscarriage Preeclampsia IUGR	Role of oxidative stress in pathophysiology
Sultana et al. [23] (Australia)	Pathophysiology review (30 publications) Total antioxidant capacity (TAC), total oxidant status (TOS), lipid peroxidation (LP)	IUGR Preeclampsia Preterm birth	Role in pathophysiology of OS and placental ageing
Jauniaux et al. [52] (France)	Pathophysiology review	Preeclampsia IUGR Spontaneous pregnancy loss Gestational diabetes mellitus (GDM)	The effect of oxidative stress on trophoblast cells
Sultana et al. [54] (USA)	*n* = 30publikacji review TAC, TOS (total oxidant status), produkty peroksydacji lipidów	Preterm birth (PTB)	Oxidative stress maybe associated with PTB

## Data Availability

Research article: Medline, Cochrane and Scopus databases

## Abbreviations

A-T: Ataxia-telangiectasia
AL: Aluminum
BS: Bloom syndrome
CAT: Catalase
CHD: Congenital heart defects
CNS: Central nervous system
DCFH: Dichlorofluorescin
DHR: Dihydrorhodamine
DM: Pregestational diabetes
eNOS: Endothelial nitric oxide synthase
FGR: Fetal growth restriction
GDM: Gestational diabetes mellitus
GSH: Glutathione
GSH-Px: Glutathione peroxidase
GSHR: Glutathione reductase
GSSG: Glutathione disulfide
GST: Glutathione S-transpherase
HE: Hydroethidine
HOBr: Subbromic acid
HOCl: Hypochlorous acid
HO2∙: Hydroperoxide radical
HPF: Fluorescein
HSP: Heat-shock protein
H2O2: Hydrogen peroxide
LDL: Low-density-lipoprotein
LP: Lipid peroxidation
MDA: Malondialdehyde
NBS: Nijmegen syndrome
NK: Natural killer
NO: Nitric oxide
OH∙: Hydroxyl radical
OS: Oxidative stress
OSI: Oxidative stress index
OX: Xanthine oxidase
O2∙: Superoxide anion radical
PB: Lead
PE: Preeclampsia
PIH: Pregnancy-induced hypertension
PON1: Paraoxonase
PPROM: Preterm prelabour rupture of membranes
PPTD: Postpartum thyroid dysfunction
PTB: Preterm birth
PUFA: Polyunsaturated fatty acids
Rcan1 gene: Regulator of calcineurin 1
RO∙: Alkoxy radical
ROO∙: Peroxide radical
ROS: Reactive oxygen species
RPL: Recurrent pregnancy loss
RSA: Recurrent spontaneous abortion
SOD: Superoxide dismutase
TAC: Total plasma antioxidant capacity
TAS: That total plasma antioxidant
TCS: Treacher Collins syndrome
TOL: Total oxidant level
TRX: Thioredoxin
XOD: Xanthine oxidase (XOD)
8-OHdG: 8-Hydroxy-2′-deoxyguanosine.
